# LT-YOLO: long-term temporal enhanced YOLO for stenosis detection on invasive coronary angiography

**DOI:** 10.3389/fmolb.2025.1558495

**Published:** 2025-04-02

**Authors:** Jiaxin Li, Xiang Tang, Xuesong Wang

**Affiliations:** ^1^ School of Biomedical Engineering, Sun Yat-sen University, Shenzhen, China; ^2^ College of Mining Engineering, University of Science and Technology Liaoning, Anshan, China

**Keywords:** coronary artery disease, stenosis detection, state-space model, Mamba, YOLO

## Abstract

Coronary artery stenosis detection by invasive coronary angiography plays a pivotal role in computer-aided diagnosis and treatment. However, it faces the challenge of stenotic morphology confusion stemming from coronary-background similarity, varied morphology, and small-area stenoses. Furthermore, existing automated methods ignore long-temporal information mining. To address these limitations, this paper proposes a long-term temporal enhanced You Only Look Once (YOLO) method for automatic stenosis detection and assessment in invasive coronary angiography. Our approach integrates long-term temporal information and spatial information for stenosis detection with state-space models and YOLOv8. First, a spatial-aware backbone based on a dynamic Transformer and C2f Convolution of YOLOv8 combines the local and global feature extraction to distinguish the coronary regions from the background. Second, a spatial–temporal multi-level fusion neck integrates the long-term temporal and spatial features to handle varied stenotic morphology. Third, a detail-aware detection head leverages low-level information for accurate identification of small stenoses. Extensive experiments on 350 invasive coronary angiography (ICA) video sequences demonstrate the model’s superior performance over seven state-of-the-art methods, particularly in detecting small stenoses (<50%), which were previously underexplored.

## 1 Introduction

**FIGURE 1:**
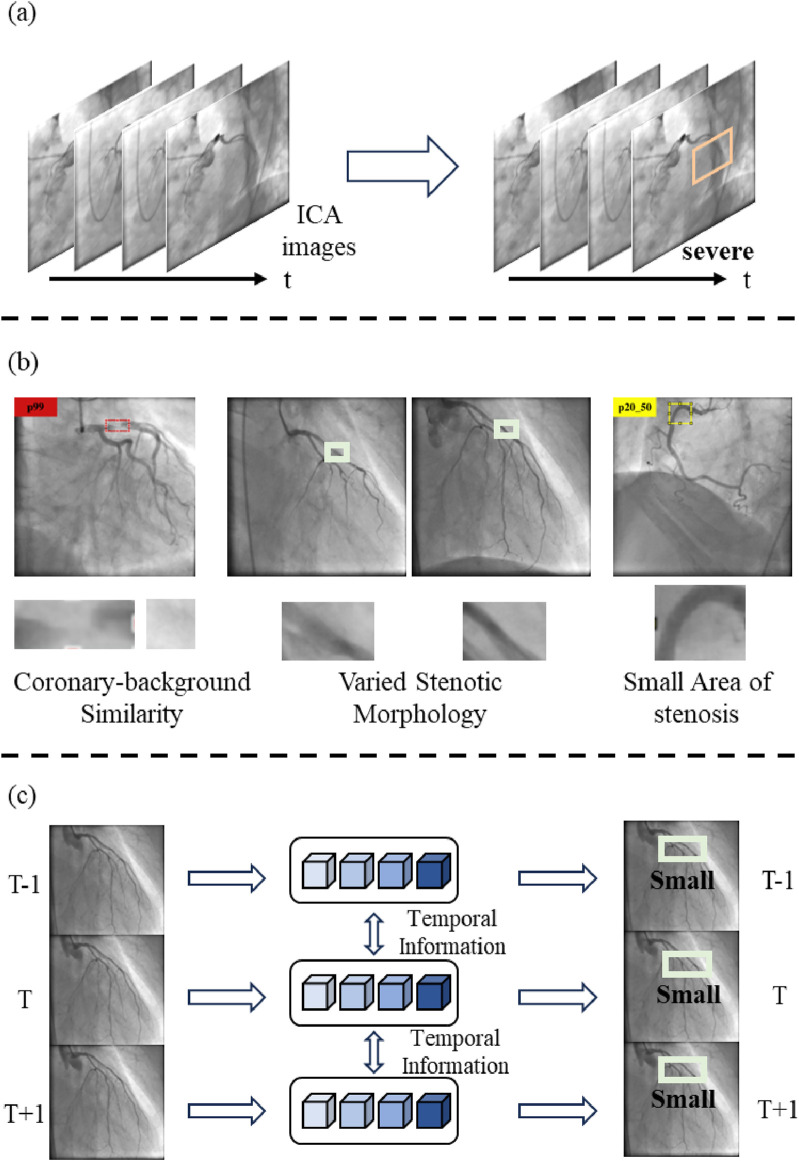
The contribution of LT-YOLO. **(A)**The process of coronary stenosis detection and assessment. **(B)**LT-YOLO addresses the challenges of stenotic morphology confusion, such as the coronary-background similarity, varied stenotic morphology, and small-area stenoses. **(C)**This paper proposes a method that embeds long-term temporal information with state-space models into the object detection pipeline.

Invasive coronary angiography is collected with the X-ray cardiovascular angiography equipment. Coronary artery stenosis detection is a crucial task in computer-aided diagnosis and coronary artery disease (CAD) treatment. CAD, resulting from the accumulation of the inner wall’s atherosclerotic plaque of the coronary artery (Lu et al., 2021), is a leading cause of death worldwide (Tsao et al., 2023). In high-income countries, it accounts for approximately one-third of total deaths (Bauersachs et al., 2019). The stenosis severity provides a basis for appropriate clinical treatment strategies for CAD. Invasive coronary angiography (ICA) has been utilized to assess stenosis severity (Garrone et al., 2009). It displays the coronary arteries with X-ray cardiovascular angiography equipment, allowing clinicians to evaluate and determine whether coronary stenosis is present. However, traditional visual assessment of the degree of stenosis relies on experienced clinicians. This process is time-consuming and subjective (Wu et al., 2020). Furthermore, the visual assessment tends to focus on severe stenoses and ignores stenoses of less than 50%, which are also meaningful for a CAD diagnosis (Jiménez-Partinen et al., 2024). Therefore, automatic detection of stenoses in ICA images is of utmost importance in the diagnosis and treatment of CAD. The stenosis detection process is shown in Figure 1A.

Stenotic morphology confusion poses challenges to stenosis detection in the ICA images. Figure 1B visually illustrates these challenges. First, the contrast between the coronary artery and the background is low. The low contrast is caused by an insufficient amount of contrast agent and the limited power in the X-ray (Li et al., 2024). It leads to confusing the coronary artery with the background. This confusion hinders stenosis detection and stenosis severity assessment. Second, the stenotic morphology is varied. The different views and the heartbeat movements result in different stenotic morphology (Pang et al., 2021). These factors lead to the misdetection of the stenosis. Third, the area of the stenosis is small with respect to the whole image. The small area makes it easy to lose the details of the stenosis and hinders the assessment of the stenosis severity, especially when the stenosis percentage is less than 50%.

Current stenosis detection methods can be categorized into two types: one focusing on single-frame images (Compas et al., 2014; Mohan and Vishnukumar, 2016; Wan et al., 2018; Kishore and Jayanthi, 2019; Au et al., 2018; Danilov et al., 2021; Cong et al., 2019a; Zhang et al., 2022) and the other on temporal image sequences (Wu et al., 2020; Pang et al., 2021; Zhang et al., 2019). For single-frame approaches, some methods (Compas et al., 2014; Mohan and Vishnukumar, 2016; Wan et al., 2018; Kishore and Jayanthi, 2019; Au et al., 2018) detect stenosis by utilizing vessel diameters. These approaches first extract the vessels, calculate diameter variations, and then locate stenotic regions based on these changes. However, such methods impose strict requirements on the accuracy of vessel boundary segmentation.

Other single-frame approaches directly perform localization and stenosis classification on cross-community access (XCA) images. For instance, Du et al. (2018) used a multi-level convolutional neural network to extract texture features at different levels for stenosis detection and localization. Similarly, Cong et al. (2019a) employed a combination of convolutional neural networks and recurrent neural networks to select key frames and classify coronary artery stenosis. However, a single-frame image carries limited information. In particular, the stenosis is moving in each frame, and it is difficult to assess the full picture of the stenosis at a single time point. Temporal information helps better observe and understand the stenosis, so many methods that utilize temporal context have been proposed. The dynamic information of the stenosis in a time series helps comprehensively observe the stenosis from various morphological changes. For example, Zhang et al. (2019) extracted sequential temporal features using a 3D convolutional neural network and an attention mechanism to assist in stenosis detection from keyframe images. Pang et al. (2021) extract feature maps frame by frame from the sequence, use an attention mechanism to fuse sequential features, and decode the output to generate stenosis detection boxes.

However, these methods ignore the long-term temporal information, such as the changes in the whole video. While short-term temporal information can partially mitigate these issues by tracking vessel state changes, persistent noise may be incorrectly interpreted as normal vessel behavior. This misinterpretation compromises detection reliability. In contrast, long-term temporal information can obtain precise vessel structure information by capturing changes in vessels and their environment over an extended period. Some methods, such as long short-term memory networks (LSTMs) (Cong et al., 2019b; Cong et al., 2023; Rodrigues et al., 2021) and recurrent neural networks (RNNs) (Fischer et al., 2020), can extract temporal information to a certain extent, but when the sequence becomes longer, it is easy to forget long-distance information, and performance will decrease (Qin et al., 2024). At the same time, LSTM is also susceptible to noise in the sequence (Qin et al., 2023). The lack of such long-term temporal information makes it difficult for the model to remove noise from the complex temporal changes in ICA videos and extract more stable and generalized features. These limitations hinder the effectiveness of these methods for stenosis detection and evaluation.

Recently, Mamba based on state-space models has been researched as an effective and economical method for modeling long-term sequences (Gu and Dao, 2023; Zhang et al., 2024). Mamba excels at capturing complex dependencies in sequential data and its data-dependent state parameters also allow for flexible state modeling. Moreover, Mamba can model sequences in linear time. This efficiency surpasses other sequence modeling methods (Dang et al., 2024). Mamba shows great potential for embedding long-term temporal information into the stenosis detection pipeline.

This article proposes a long-term temporal enhanced You Only Look Once (YOLO) (LT-YOLO) method for stenosis detection and assessment on invasive coronary angiography (shown in Figure 1C). LT-YOLO combines long-term temporal information and multi-level spatial information through state-space models to identify diverse stenotic morphology. It is based on a YOLOv8 structure and realizes the following improvements: First, a spatial information perception backbone is designed. This backbone replaces the last layer of the YOLOv8 backbone with our carefully designed dynamic transformer block. The C2f structure of the YOLOv8 backbone gains great performance on local information extraction, while the dynamic transformer block utilizes our dual-stream self-attention mechanism to flexibly extract the context and structural features of the images. The combination of the two parts enables the model to better distinguish between the coronary artery and the background semantics. Second, a spatial–temporal multi-level fusion neck is designed. This neck fuses temporal and spatial information at multiple feature levels. It conveys long-term temporal information among each feature level through the state-space model. Then, the PANet in YOLOv8 conveys spatial information across different levels. This multi-level spatial-temporal information fusion mechanism enables the model to perceive the features of the stenosis regions from multiple dimensions, thus handling the varied stenotic morphology. Third, a detail-aware detection head is designed. This head utilizes the feature from the first layer to convey low-level information into the head with a cross-attention mechanism. This low-level information helps the head identify details of the stenosis and handle the small stenosis regions. Our contributions can be summarized as follows:

1. This paper describes an automatic tool for stenosis detection and assessment in invasive coronary angiography to assist the workflow of the computer-aided diagnosis;
2. This paper proposes to mine long-term temporal information for stenosis detection, which has been ignored in the previous research;
3. This paper inspects the stenotic morphology confusion problem in the stenosis detection task and solves it in the backbone, neck, and detection head of the YOLO;
4. Extensive experiments on 350 ICA video images show that LT-YOLO achieves superior stenosis detection compared to seven state-of-the-art methods. Its performance is especially good when detecting stenoses of less than 50%, which have been ignored by the previous methods.

## 2 Related work

### 2.1 Automatic detection of coronary artery stenosis

The detection and evaluation of coronary artery stenosis is a classic problem in the field of automated cardiovascular disease assessment. With the rapid development of artificial intelligence, an increasing number of studies have employed ICA image data for automated analysis. By leveraging computer vision and object detection methods, stenosis locations and types can be identified quickly and objectively, aiding in diagnostic analysis.

Early methods for automatic stenosis detection primarily relied on comparing variations in vessel radius. For example, Compas et al. (2014) calculated vessel diameters based on image intensity changes, generating a vessel diameter surface where the minimum value corresponded to the stenotic region. Wan et al. (2018) applied image enhancement techniques and extracted vessel skeletons using the level set algorithm. Subsequently, the vessel radius and orientation were calculated, and local extrema were used to identify stenotic locations. Coronary artery segmentation results are often utilized to extract vessel diameters, which are then used to detect and classify stenosis. However, such methods (Mohan and Vishnukumar, 2016; Kishore and Jayanthi, 2019; Au et al., 2018) heavily depend on the accurate extraction of vascular structures, making it challenging to achieve reliable and consistent stenosis detection.

With the development of neural networks, some end-to-end methods have been directly applied to stenosis detection. Ovalle-Magallanes (2022) combined convolutional neural networks (CNN) and quantum networks to directly extract stenotic regions from single-frame images. Du et al. (2018) used multi-level CNNs to extract features of different sizes from images and then performed stenosis detection and localization. Cong et al. (2019a) employed a combination of CNN and recurrent neural networks (RNNs) to first select key frames for stenosis and then classify coronary artery stenosis.

Single-frame-based methods struggle to address issues such as vessel deformation caused by respiratory and cardiac motion, vessel occlusion, and limited foreground-background differences. Temporal information can be used more comprehensively to evaluate stenosis. Zhang et al. (2019) first used two 3D CNNs to integrate temporal information from angiographic sequences at two angles and determined the severity of stenosis after merging features with an attention mechanism. Wu et al. (2020) used temporal constraints to reduce false positives. However, these constraints are highly sensitive to vessel movement. Pang et al. (2021) performed stenosis detection frame by frame in the sequence and then fused features from candidate boxes in these frames, optimizing the initial detection boxes. However, subsequent feature fusion depends on the results of the initial single-frame detection. Han et al. (2023) proposed a spatiotemporal feature aggregation module, which extracts features from local regions of interest and aggregates them using an attention mechanism for stenosis detection. However, the computational demands of the attention mechanism and the proposal of regions of interest significantly slow down inference speed. In summary, while the methods combining temporal information have improved stenosis detection, they do not explicitly model long-term temporal context and thus struggle to globally understand dynamic evolution. The reliance on local temporal information may limit the effectiveness of these methods in stenosis detection and assessment.

### 2.2 Applications of Mamba to computer vision

Mamba (Gu and Dao, 2023) is a selective structured state-space model (SSM), where the state-space model serves as a system for mapping sequential data. It maps inputs to latent state variables and generates outputs through the evolution of these states. Due to its global receptive field and linear complexity, Mamba has gained considerable attention in computer vision tasks.

Ma et al. (2024) proposed a U-shaped network combining convolutional neural networks and Mamba for biomedical image segmentation, which enhances long-term dependency in images. Zhu et al. (2024) introduced bidirectional scanning Mamba blocks, a computationally efficient and general-purpose vision backbone. Yang et al. (2024) proposed temporal Mamba blocks using multi-directional scanning to model spatiotemporal dependencies in video sequences. Shi et al. (2024) developed a multi-level Mamba model to enhance the influence of long-term information.

Other works have applied Mamba in medical image analysis (Xing et al., 2024; Ye et al., 2024; Hao J. et al., 2024; Liu et al., 2024; Ruan and Xiang, 2024). For example, Hao et al. (2024a) introduced frequency-domain features into Vision Mamba to improve the performance of low-contrast cone beam computed tomography segmentation. Liu et al. (2024) enhanced the performance of Mamba in medical image segmentation networks by using models pre-trained on natural datasets. Ruan and Xiang (2024) also integrated Mamba into the U-shaped network structure for medical image segmentation, improving computational efficiency.

### 2.3 Applications of YOLO in medical imaging

You Only Look Once (YOLO) (Redmon et al., 2016) is an object detection algorithm that uses convolutional neural networks to detect regions of interest in real time. It splits an image into a grid of cells, and each cell is in charge of detecting objects in a particular region. It is faster than the traditional two-stage methods, which makes it applicable to real-time scenarios. YOLO has undergone several iterations and upgrades since its initial proposal (Ragab et al., 2024), overcoming limitations and improving performance. Its remarkable performance has garnered widespread attention and application across various fields.

In medical image processing, YOLO is mainly applied to the detection and localization of anatomical structures (Mortada et al., 2023; Zeng et al., 2023), lesions (Baccouche et al., 2021; Santos et al., 2022), tumors (Montalbo, 2020), and other regions of interest (Zhou et al., 2023). YOLO helps enhance diagnostic accuracy and facilitates more effective treatment processes. YOLO has strong detection capabilities across various modalities of medical images, including X-rays (Hao S. et al., 2024; Adji et al., 2021), MRI scans (Almufareh et al., 2024; Rahimi et al., 2024), ultrasound images (Wang et al., 2023; Cao et al., 2019), and CT scans (Ji et al., 2023; Liu, 2022). It achieves high detection accuracy for conditions such as lung nodules (Liu, 2022), breast nodules (Hao S. et al., 2024), vascular stenosis (Wang et al., 2024), and tumors (Rahimi et al., 2024). YOLO has been successfully applied in the segmentation of organs such as the heart (Balasubramani et al., 2024), liver (Randar et al., 2024), and other organs (Hammami et al., 2020). Precise organ segmentation is crucial for disease assessment and surgical planning.

YOLO also serves as a valuable tool for computer-assisted diagnosis (Wang et al., 2022; Amiri Tehrani Zade et al., 2023). It can detect and track surgical instruments and other regions of interest in real time during surgery. This ability helps surgeons quickly identify targets, plan surgical paths, and ultimately improves the safety and efficiency of surgeries.

## 3 Methods

The study designs the LT-YOLO to detect stenosis and assess its severity. As a whole, the LT-YOLO embeds three novel modules into the YOLOv8 structure. The three modules aim to handle stenotic morphology confusion and detect small, moderate, and severe stenosis accurately.

Specifically, the input of the network is a sequence of frames $X=\left\{x_{0},x_{1},\ldots ,x_{n}\right\}$ . The output is the prediction result set of the sequence: $R=\left\{\left(r_{0,d},r_{0,a}\right),\left(r_{1,d},r_{1,a}\right),\ldots ,\left(r_{n,d},r_{n,a}\right)\right\}$ , where $r_{i,d}$ denotes the detection result of the i -th frame, and $r_{i,a}$ denotes the severity assessment result of the i -th frame.

### 3.1 Spatial-aware backbone

**FIGURE 2:**
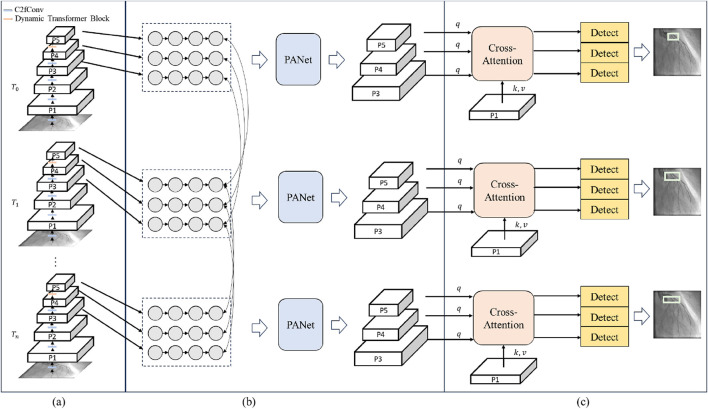
The structure of LT-YOLO. **(A)** *T_0-T_n denote the time steps of the input sequence*. P1-P5 denote different levels of the feature maps. C2fConv denotes the standard convolution layer of YOLOv8. Dynamic Transformer block denotes the block proposed in this article. **(B)** PANer denotes the feature fusion process in YOLOv8. **(C)** q, k and v denote the query, key and value of the cross-attention mechanism. Detect denotes the detection head of the model.

**FIGURE 3:**
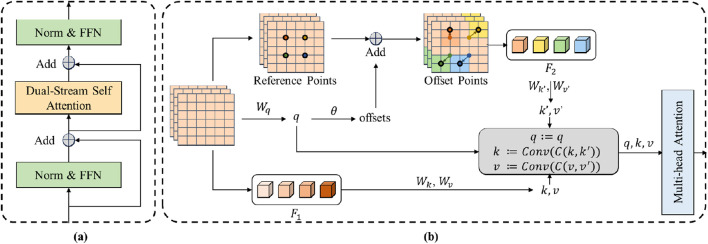
The structure of the dynamic Transformer block. **(A)** The Norm&FFN denotes the process of the normalization and the feed forward network. Add denotes the element-wise addition operation of the feature maps. **(B)** F1 and F_2 denotes the sampled features of the input feature map and the deformable feature map, respectively. q, k, v denote the query, value and key of the standard self-attention. q’, k’, v’ denote the query, value and key of the deformable self-attention. Wq, Wk, W_v Wq’, Wk’ and W_v’ denote the projection matrices of the queries, keys and values. θ denotes the function for calculating the offset of the reference points. Conv denotes the convolution layer that combines the keys and values.

The spatial-aware backbone is designed to extract the spatial information of each frame. The structure of a spatial-aware backbone is shown in Figure 2A. It replaces the last layer of the YOLOv8 backbone with the dynamic transformer block. The spatial-aware backbone can be divided into several stages. The C2f convolution is utilized to extract the low-level features f . Then, f is fed into the dynamic transformer block (shown in Figure 3A) using Equation 1:

$$\begin{array}{cc} A_{i}^{j} & \;=s\left(l\left(f_{i}^{j}\right)\right)+x\\ Y_{i}^{j} & \;=MLP\left(l\left(f_{i}^{j}\right)\right)+A_{i}^{j} \end{array},$$ (1)

where x denotes the input feature map of the transformer block. l denotes the layer norm. s denotes the core of our dynamic transformer block–the dual-stream self-attention (shown in Figure 3B). MLP denotes the multi-layer perception.

The dual-stream self-attention can achieve dynamic self-attention based on the semantic relationships in each frame of ICA sequences to distinguish between the coronary arteries and the background. The challenge of distinguishing stenotic regions stems from their diverse feature presentations and similarities to background coronary artery tissue. The dual stream combines the regular and deformable self-attention to extract comprehensive semantic context features. These semantic context features help to improve this distinguishing ability (Sun et al., 2023). As the core of the transformer, the self-attention mechanism is a widely known method for extracting context information (Vaswani, 2017). However, the regular self-attention mechanism is restricted by the fixed patch partitioning mechanism. It cannot flexibly allocate attention positions for different input images, thus failing to obtain accurate semantic information. Deformable self-attention is a mechanism that flexibly allocates attention positions (Xia et al., 2022). It learns several groups of offsets that are independent of the query to shift the key and value to important regions, ensuring different responses to different image regions. The dual-stream self-attention combines the regular self-attention and the deformable self-attention, enabling the module to generate semantic features that take both global and object-specific information into account. This complementary approach significantly enhances the model’s ability to differentiate between stenotic and non-stenotic areas by leveraging rich contextual information that singular attention mechanisms might miss.

The structure of the dual-stream self-attention is shown in Figure 3B. Specifically, the dual-stream self-attention sees the deformable self-attention and the regular self-attention as two parallel branches and generates keys and values, respectively. The two groups of key-value are concatenated in dimension and then subjected to dimension reduction of feature dimensions through a Convolution layer. The concentrated key value is utilized to process the queries.

The dual-stream self-attention can be denoted as Equation 2:

$$\begin{array}{c} K,V=Conv\left(C\left(fW_{k},f^{\prime }W_{k}\right)\right),Conv\left(C\left(fW_{v},f^{\prime }W_{v}\right)\right)\\ \;Q=fW_{q}\\ \;a^{m}=\sigma \left(\frac{Q^{m}K^{m^{T}}}{\sqrt{d_{k}}}\right)V^{m},\;m=1,\ldots ,M\\ \;A=C\left(z^{1},\ldots ,z^{M}\right)W_{f} \end{array},$$ (2)

where Q , K , and V denote the query, key, and value of self-attention. f denotes the input of the dual-stream self-attention. $f^{\prime }$ denotes the deformable feature map. $W_{q}$ , $W_{k}$ , and $W_{v}$ denote the projection matrices for Q , K , and V . $W_{f}$ denotes the projection matrix for the output. $a^{m}$ denotes the output of the m -th attention head. A denotes the output of the multi-head attention. σ denotes the sigmoid function. C denotes the concatenation. Conv denotes the convolution layer.

The deformable feature map $f^{\prime }$ is generated using Equation 3

$$\begin{array}{cc} \;x^{\prime }=\Phi \left(f;p+\Delta p\right)\\ \;p=\left\{\left(i^{\prime },j^{\prime }\right)|i^{\prime }=\frac{2i}{h/r-1},j^{\prime }=\frac{2j}{W/r-1}\right\}\\ \text{where}\; & \;i,j\in \left\{x|x\in N,0\leq x\leq \frac{H}{r}-1\right\}\\ \;\Delta p=\theta \left(Q\right) \end{array}$$ (3)

The format of this equation seems incorrect. where p denotes the reference point set. H and W denote the height and width of f . r denotes the distances between each point. θ denotes the function for calculating the offset of the reference points. Specifically, θ is set as a network with a DWConv layer for estimating the offset and a 1∗1 Convolution layer for reducing the feature dimension. The sampling function Φ(⋅,⋅) is set as Equation 4:

$$\phi \left(f;p\right)=\underset{\left(r_{x},r_{y}\right)}{\sum }\max\left(0,1-|p_{x}-r_{x}|\right)\max\left(0,|1-p_{y}-r_{y}|\right)f\left[r_{y},r_{x};\right],$$ (4)

where $\left(r_{x},r_{y}\right)$ denotes the indexes of the locations on f .

### 3.2 Multi-level spatial–temporal fusion module

The multi-level spatial-temporal fusion module aims at embedding temporal information into the object detection pipeline. The structure of the multi-level spatial-temporal fusion module is shown in Figure 2A. Specifically, it splits the feature map from each level into several patches and sees each patch as a state. The temporal information is conveyed through the Mamba within each level. Then, the spatial information is aggregated across different levels.

The process of conveying temporal information can be denoted as Equation 5:

$$\begin{array}{cc} F^{i} & \;\;=\text{Mamba}\left(x^{i}\right)\\ x^{i} & \;\;=\left\{S_{T_{0}}^{i,P_{0}},S_{T_{0}}^{i,P_{1}},\ldots ,S_{T_{0}}^{i,P_{n}},\ldots ,S_{T_{n}}^{i,P_{0}},S_{T_{n}}^{i,P_{1}},\ldots ,S_{T_{n}}^{i,P_{n}}\right\}\\ S^{i} & \;\;=\text{PatchEmd}\left(f^{i}\right) \end{array},$$ (5)

where Mamba denotes the temporal Mamba. PatchEmd denotes the patch embedding process. $x^{i}$ denotes the generated sequence of the feature map from the i -th level.

**FIGURE 4:**
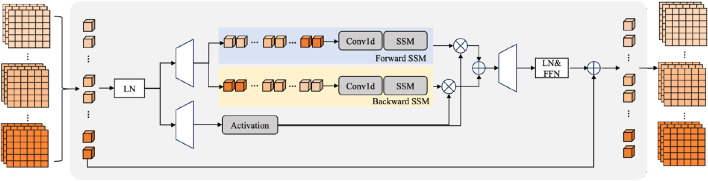
The details of the temporal Mamba.

The structure of the temporal Mamba is shown in Figure 4. The state-space models can be denoted as Equation 6:

$$\begin{array}{cc} & h_{t}=\bar{A}h_{t-1}+\bar{B}x_{t}\\ & \text{SSM}\left(x_{t}\right)=\bar{C}h_{t} \end{array}$$ (6)

where x denotes the input sequence. $h_{t}$ denotes the state in time t . $\bar{A},\bar{B}$ and $\bar{C}$ denote the parameters of the state-space models.

In LT-YOLO, the bidirectional SSM is utilized to comprehensively mine the long-term information. The forward SSM and backward SSM can be denoted as Equation 7:

$$\begin{array}{cc} {\text{SSM}}^{f}\left(X\right) & \;=\text{SSM}\left(\text{Conv}\left(X\right)\right)\\ {\text{SSM}}^{b}\left(X\right) & \;=\text{SSM}\left(\text{Conv}\left(\text{Inv}\left(X\right)\right)\right) \end{array},$$ (7)

where Inv(⋅) denotes the inverse function.

Then, the spatial information is fused across each level through the PANet in YOLOv8 (Sohan et al., 2024).

### 3.3 Detail-aware detection head

The detail-aware detection head is based on the improved YOLOv8 head. It extracts the detailed information from the low-level feature map to enhance the structural information absent in the high-level features. It is beneficial for detecting small stenosis regions. To achieve this purpose, the detail-aware detection head utilizes a cross-attention mechanism between the low-level features and the high-level feature maps (Shim et al., 2023). The structure of the detail-aware detection head is shown in Figure 2C.

Specifically, the detail-aware detection head utilizes $F^{1}$ to generate the key and value, and the $F^{3},F^{4},F^{5}$ as the queries to implement the cross-attention mechanism. The process can be denoted as Equation 8:

$$f_{ca}\left(Q,K,V\right)=\text{softmax}\left(\frac{QK^{T}}{\sqrt{d_{k}}}\right)V,$$ (8)

where $f_{ca}$ denotes the cross-attention function, and $d_{k}$ is the dimensionality of the key. In this module, Q,K,V can be defined as Equation 9:

$$\begin{array}{cc} K,V\; & =f_{pl}\left(f_{LN}\left(F^{1}\right),d_{k},d_{k}\right)\\ Q_{i} & =f_{pl}\left(f_{LN}\left(F^{i}\right),d_{k},d_{k}\right) \end{array},$$ (9)

where i=3,4,5 , $f_{LN}$ is the linear norm, and $f_{pl}$ is the linear projection. Then, $F^{3},F^{4},F^{5}$ are decoded to the boundary-enhancement feature map using Equation 10:

$$\begin{array}{c} {\text{CA}}_{i}=f_{ca}\left(Q_{i},K,V\right)\\ P^{i}=f_{\mathrm{FFN}}\left(f_{LN}\left({\text{CA}}_{i}+F^{i}\right)\right)+{\text{CA}}_{i}+F^{i} \end{array},$$ (10)

where $F_{\mathrm{FFN}}$ denotes the feed forward network.

Then, the decoded multi-level features are fed into the detection head to obtain the final prediction boxes.

## 4 Experiments

### 4.1 Dataset, experimental setup, and evaluation metrics

#### 4.1.1 Dataset and experimental setup

**FIGURE 5:**
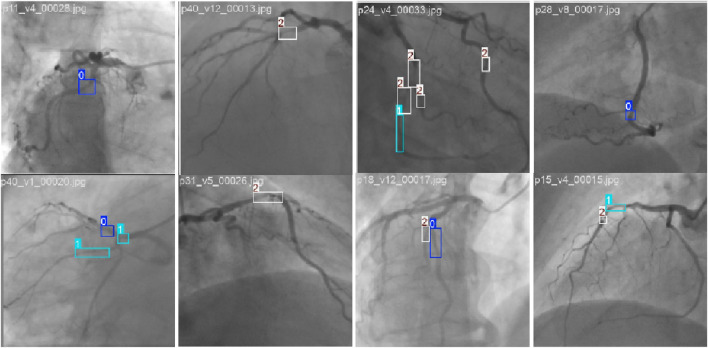
Example of stenosis detection and assessment images in the CADICA dataset. The 0, 1, and 2 denote the p0-50, p50-70, and p70-100 stenoses, respectively.

**FIGURE 6:**
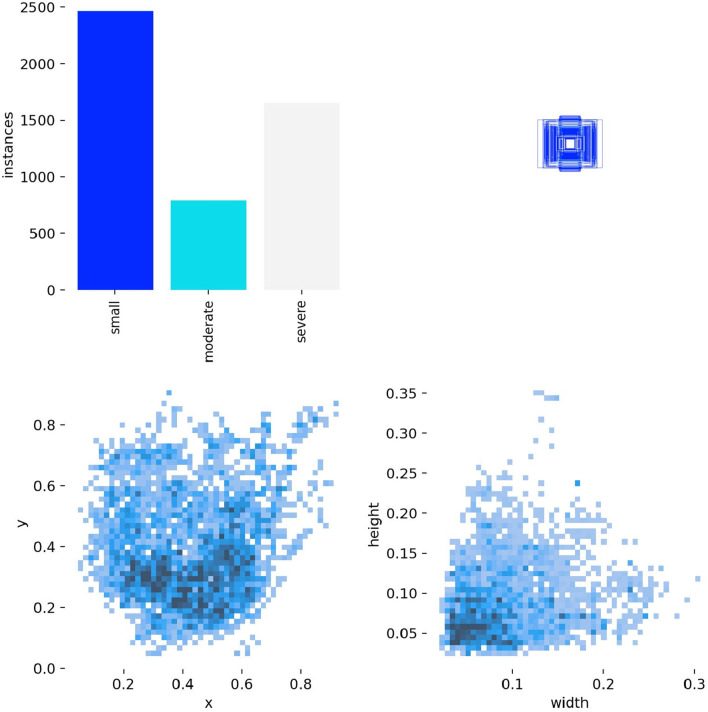
The label distribution of our dataset.

The experiments are performed on 350 videos extracted from the Coronary Angiography Digital Imaging and Communication Archive (CADICA) (Jiménez-Partinen et al., 2024). CADICA is a public dataset composed of ICA videos of 42 patients. The annotations of the dataset are in the format of (c, x, y, w, h) of the boxes that surround the stenosis regions. The c denotes the class of the stenosis, which is divided into three categories according to the stenosis percentage: <50% (p0-50, small), 50%–70% (p50-70, moderate), and >70% (p70-100, severe). Figure 5 shows the example images of our dataset. Figure 6 shows the label distribution of our dataset, from which 350 videos are sampled. Each video is composed of 10 consecutive contrast-filled frames. For the dataset composition, the diversity in sequence selection is ensured by incorporating various vascular patterns and stenosis degrees. Furthermore, for each patient, multiple viewing perspectives are sampled to capture anatomical variations. These selection strategies ensure the maximum data representativeness. The 5-fold cross-validation is implemented, with three folds for training, one for validation, and one for testing. The final results reported in the manuscript represent the average performance across all folds. The learning rate (lr) is configured to 1e−2. The momentum parameter is adjusted to 9e−1 with the weight decay configured to 1e−4. During the experiment, each frame is resized into 512×512. During training, all experimental methods utilize identical data augmentation strategies: flipping, brightness adjustment, and copy–paste operations. The hardware environment utilized in this experiment is NVIDIA RTX A6000.

#### 4.1.2 Evaluation metrics

The average position (AP) of different classes and the mean average position (mAP) at 0.5 are used to evaluate the detection result of LT-YOLO. The four metrics are calculated through Equation 11:

$$\begin{array}{c} \;\text{P}=\frac{\text{TP}}{\text{TP}+\text{FP}}\\ \;\text{R}=\frac{\text{TP}}{\text{TP}+\text{FN}}\\ \;\text{AP}=\int_{0}^{1}\text{P}d\text{R}\\ \text{mAP}=\frac{\sum_{i=1}^{K}{\text{AP}}_{i}}{K} \end{array},$$ (11)

where TP denotes the true positive instances (correctly detected objects). FP denotes the false positive instances (incorrectly detected objects). FN denotes the false negative instances (objects not detected). P denotes the precision. R denotes the recall. K denotes the num of the classes.

The frames per second (FPS) value is utilized to assess the inference time. The FPS is calculated with Equation 12:

$$FPS=\frac{N}{t},$$ (12)

where N represents the quantity of frames, and t is the processing time measured in seconds.

### 4.2 Experimental results and analysis

**FIGURE 7:**
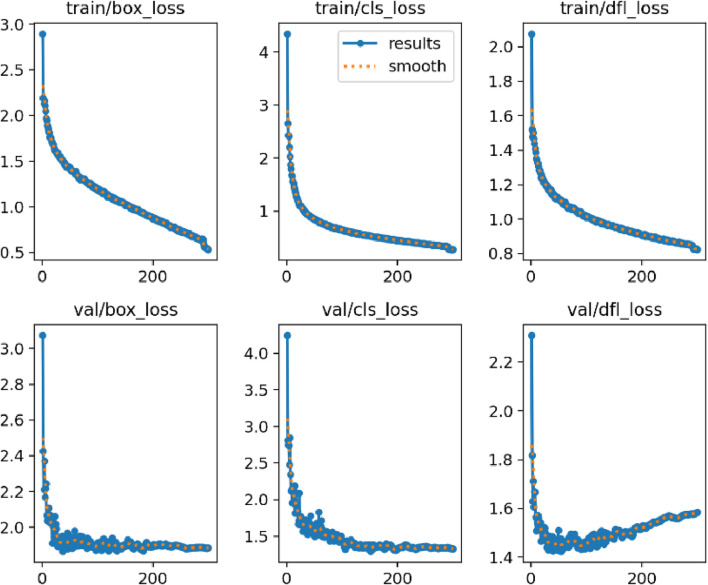
The loss curves for model training.

A comparison experiment and ablation study is conducted on our dataset to evaluate the effectiveness of LT-YOLO. The comparison experiment compares the performance of LT-YOLO with other object detection methods. The ablation study proves the effectiveness of the three components of LT-YOLO. Figure 7 illustrates the training process of LT-YOLO. The loss curves in Figure 7 indicate that both the train loss and the val loss show a trend of declining rapidly at first and then remaining relatively stable. It suggests that the training pipeline is effective with no signs of either under-fitting or over-fitting.

#### 4.2.1 Comparison experiment

A comparison experiment is conducted between LT-YOLO and the state-of-the-art object detection methods, including RetinaNet (Ross and Dollár, 2017), Faster R-CNN (Ren et al., 2016), mask R-CNN (He et al., 2017), Cascade R-CNN (Cai and Vasconcelos, 2018), YOLOv3 (Farhadi and Redmon, 2018), YOLOv5 (Jocher et al., 2022) and YOLOv8 (Sohan et al., 2024).

As shown in Table 1, LT-YOLO gives the best performance. The overall mAP increases by 2.9%–16.2%. The APs of p0-50, p50-70, and p70-100 increase by 32.6%–3.9%, 4.5%–10.6%, and 0.3%–8.5%, respectively. These results prove the superior performance of LT-YOLO against other object detection methods.

**TABLE 1:** Performance comparison experiments between LT-YOLO and other state-of-the-art object detection methods based on the stenosis detection and assessment dataset.

Model	${AP}_{p0-50}$ (%)	${AP}_{p50-70}$ (%)	${AP}_{p70-100}$ (%)	mAP@0.5 (%)
RetinaNet	37.5	69.8	73.2	60.2
Faster R-CNN	52.2	70.7	75.6	66.2
Mask R-CNN	59.1	70.5	75.1	68.2
Cascade R-CNN	61.7	71.5	70.1	67.8
YOLOv3	63.2	72.5	77.1	70.9
YOLOv5	64.5	72.9	77.8	71.7
YOLOv8	66.2	75.9	78.3	73.5
LT-YOLO	70.1	80.4	78.6	76.4

^a^ Tables may have a footer.

**FIGURE 8:**
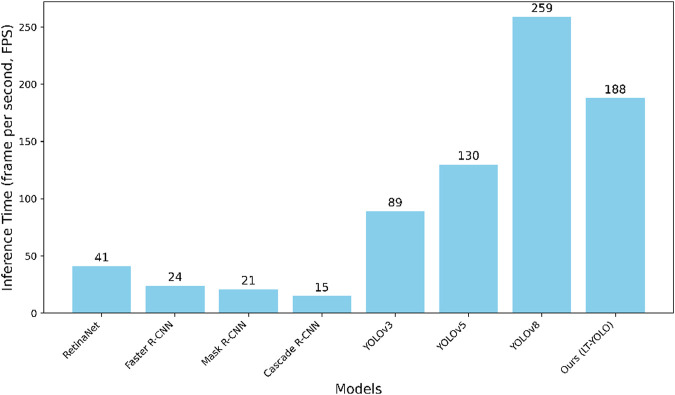
Inference time performance of LT-YOLO and other state-of-the-art methods.

**FIGURE 9:**
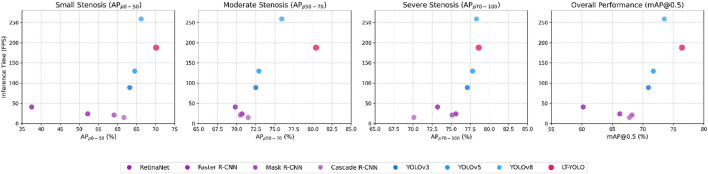
FPS-mAP relationship for the comparison experiment.

Figure 8 compares the inference time (frames per second) of all methods. Although LT-YOLO shows a slightly slower speed than YOLOv8 (which has the fastest inference time), it achieves better accuracy in coronary artery stenosis detection. Meanwhile, LT-YOLO still maintains faster inference times than all other comparison methods. This trade-off between speed and accuracy is appropriate for clinical applications where detection precision takes priority over processing speed. Figure 9 shows the FPS-mAP relationship comparison of all methods in detail. The LT-YOLO proposed in this article achieves the best detection effect with suboptimal inference time performance.

#### 4.2.2 Ablation study

To evaluate the effectiveness of the different components of LT-YOLO, an ablation study was conducted on the stenosis detection and assessment dataset. The result is shown in Table 2. A denotes the spatial-aware backbone. B denotes the multi-level spatial-temporal fusion neck. C denotes the detail-aware head. Table 2 indicates that the spatial-aware backbone increases the APs of p50-70 and p70-100 to 3.3% and 0.6%, respectively. However, it decreases the AP of p0-50 by 0.9%. This is because the dynamic transformer block in the spatial-aware backbone pays attention to global information extraction and may lose detailed information. The multi-level spatial-temporal fusion neck increases the APs of p0-50, p50-70, and p70-100 to 2.9%, 2.8%, and 0.5%, respectively. With the low-level information enhancement of the detail-aware head, LT-YOLO finally increases the mAP@50 by 2.9%. It also shows that the full model decreases the A + B model by 0.2% in ${\text{AP}}_{\mathrm{p70-100}}$ . This decrease is attributed to the minor noise introduced by the detail-aware head. The head incorporates low-level information, making the model more sensitive to the small and moderated stenosis. However, it may introduce minor noise. Such minor noise might slightly affect the model’s feature processing for the large stenosis and result in a minor decrease. However, this decrease is negligible considering the model’s overall improved performance in stenosis detection.

**TABLE 2:** Ablation study on LT-YOLO. A denotes the spatial-aware backbone. B denotes the multi-level spatial–temporal fusion neck. C denotes the detail-aware head.

Model	${AP}_{p0-50}$ (%)	${AP}_{p50-70}$ (%)	${AP}_{p70-100}$ (%)	mAP@0.5 (%)
YOLOv8	66.2	75.9	78.3	73.5
+A	65.3	79.2	78.9	74.1
+B	69.1	78.7	78.8	75.5
+A + B + C(LT-YOLO)	70.1	80.4	78.6	76.4

**FIGURE 10:**
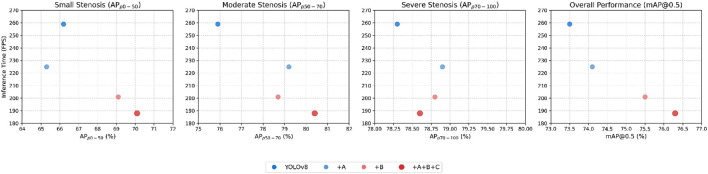
Comparison of the FPS-mAP relationship of the innovative module proposed in this paper. A denotes the spatial-aware backbone. B denotes the multi-level spatial–temporal fusion neck. C denotes the detail-aware head.

Figure 10 shows the FPS-mAP relationship comparison of all innovative models proposed in this article. The addition of the spatial-aware backbone, the multi-level spatial–temporal fusion neck, and the detail-aware head improves the accuracy to a certain extent in terms of comprehensive accuracy.

### 4.3 Visualization results

**FIGURE 11:**
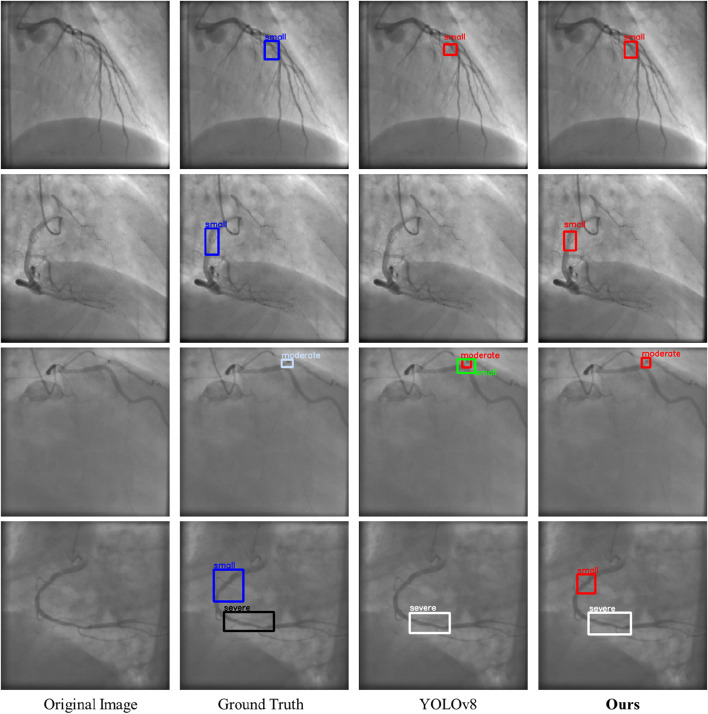
The visualization results of LT-YOLO and YOLOv8. The dark blue boxes denote the ground truth of small stenoses. The light blue boxes denote the ground truth of moderate stenoses. The black boxes denote the ground truth of severe stenoses. The red, red, and white boxes denote the prediction results of small, moderate, and severe stenosis, respectively.

Figure 11 illustrates the visualization results of LT-YOLO and YOLOv8. The four rows denote the four examples of the prediction results. In each row, the four columns denote the original image, the ground truth, the prediction result of YOLOv8, and the prediction result of LT-YOLO. The first row shows that LT-YOLO is able to locate the stenosis more accurately than YOLOv8. The second row and the last row indicate that LT-YOLO can recognize stenosis that is ignored by YOLOv8. The third row shows that LT-YOLO is more confident in stenosis assessment. In conclusion, Figure 11 proves the superior stenosis detection and assessment ability of LT-YOLO.

## 5 Conclusion

This study proposes a long-term temporal enhanced YOLO (LT-YOLO) for stenosis detection and assessment in invasive coronary angiography (ICA). LT-YOLO combines the strengths of a spatial-aware backbone, a multi-level spatial–temporal fusion neck, and a detail-aware detection head to mine long-term temporal information for addressing the challenge of stenotic morphology confusion. The LT-YOLO effectively enhances the detection of the stenosis regions and achieves superior performance compared to existing state-of-the-art methods on 350 ICA videos. While these results are promising, future work should focus on multi-center validation to evaluate the model’s performance across different patient populations and imaging protocols. This broader validation would provide comprehensive evidence for the clinical applicability of this valuable tool in aiding CAD diagnosis.

## Data Availability

The original contributions presented in the study are included in the article/supplementary material; further inquiries can be directed to the corresponding author.

## References

[B1] Adji W. A. Amalia A. Herriyance H. Elizar E. (2021). “Abnormal object detection in thoracic X-ray using you only look once (YOLO),” in 2021 International Conference on Computer System, Information Technology, and Electrical Engineering COSITE, Banda Aceh, Indonesia, 20-21 October 2021 (IEEE), 118–123.

[B2] Almufareh M. F. Imran M. Khan A. Humayun M. Asim M. (2024). Automated brain tumor segmentation and classification in MRI using YOLO-based Deep Learning. IEEE Access 12, 16189–16207. 10.1109/access.2024.3359418

[B3] Amiri Tehrani Zade A. Jalili Aziz M. Majedi H. Mirbagheri A. Ahmadian A. (2023). Spatiotemporal analysis of speckle dynamics to track invisible needle in ultrasound sequences using convolutional neural networks: a phantom study. Int. J. Comput. Assisted Radiology Surg. 18, 1373–1382. 10.1007/s11548-022-02812-y 36745339

[B4] Au B. Shaham U. Dhruva S. Bouras G. Cristea E. Md A. L. (2018). Automated characterization of stenosis in invasive coronary angiography images with convolutional neural networks. arXiv Prepr. arXiv:1807.10597. 10.48550/arXiv.1807.10597

[B5] Baccouche A. Garcia-Zapirain B. Olea C. C. Elmaghraby A. S. (2021). Breast lesions detection and classification via YOLO-based fusion models. Comput. Mater. and Continua 69, 1407–1425. 10.32604/cmc.2021.018461

[B6] Balasubramani M. Sung C. W. Hsieh M. Y. Huang E. P. C. Shieh J. S. Abbod M. F. (2024). Automated left ventricle segmentation in echocardiography using YOLO: a deep learning approach for enhanced cardiac function assessment. Electronics 13, 2587. 10.3390/electronics13132587

[B7] Bauersachs R. Zeymer U. Brière J. B. Marre C. Bowrin K. Huelsebeck (2019). Burden of coronary artery disease and peripheral artery disease: a literature review. Cardiovasc. Ther. 2019, 8295054. 10.1155/2019/8295054 32099582 PMC7024142

[B8] Cai Z. Vasconcelos N. (2018). “Cascade r-cnn: delving into high quality object detection,” in Proceedings of the IEEE conference on computer vision and pattern recognition, Salt Lake City, UT, USA, 18-23 June 2018, 6154–6162. 10.1109/cvpr.2018.00644

[B9] Cao Z. Duan L. Yang G. Yue T. Chen Q. (2019). An experimental study on breast lesion detection and classification from ultrasound images using deep learning architectures. BMC Med. imaging 19, 51–59. 10.1186/s12880-019-0349-x 31262255 PMC6604293

[B10] Compas C. B. Syeda-Mahmood T. McNeillie P. Beymer D. (2014). “Automatic detection of coronary stenosis in X-ray angiography through spatio-temporal tracking,” in 2014 IEEE 11th international symposium on biomedical imaging (ISBI), Beijing, China, 29 April 2014 - 02 May 2014 (IEEE), 1299–1302.

[B11] Cong C. Kato Y. Vasconcellos H. D. Lima J. Venkatesh B. (2019a). “Automated stenosis detection and classification in x-ray angiography using deep neural network,” in 2019 IEEE international conference on bioinformatics and biomedicine (BIBM), San Diego, CA, USA, 18-21 November 2019 (IEEE), 1301–1308.

[B12] Cong C. Kato Y. Vasconcellos H. D. Lima J. Venkatesh B. (2019b). Automated stenosis detection and classification in X-ray angiography using deep neural network. IEEE Int. Conf. Bioinforma. Biomed. (BIBM) 2019, 1301–1308. 10.1109/BIBM47256.2019.8983033

[B13] Cong C. Kato Y. Vasconcellos H. D. D. Ostovaneh M. R. Lima J. A. Ambale-Venkatesh B. (2023). Deep learning-based end-to-end automated stenosis classification and localization on catheter coronary angiography. Front. Cardiovasc. Med. 10, 944135. 10.3389/fcvm.2023.944135 36824452 PMC9941145

[B14] Dang T. D. Q. Nguyen H. H. Tiulpin A. (2024). LoG-VMamba: local-global vision mamba for medical image segmentation. Proc. Asian Conf. Comput. Vis., 548–565. 10.1007/978-981-96-0901-7_14

[B15] Danilov V. V. Klyshnikov K. Y. Gerget O. M. Kutikhin A. G. Ganyukov V. I. Frangi A. F. (2021). Real-time coronary artery stenosis detection based on modern neural networks. Sci. Rep. 11, 7582. 10.1038/s41598-021-87174-2 33828165 PMC8027436

[B16] Du T. Liu X. Zhang H. Xu B. (2018). “Real-time lesion detection of cardiac coronary artery using deep neural networks,” in 2018 International Conference on Network Infrastructure and Digital Content (IC-NIDC), Guiyang, China, 22-24 August 2018 (IEEE), 150–154.

[B17] Farhadi A. Redmon J. (2018). Yolov3: an incremental improvement, 1804. Berlin/Heidelberg, Germany: Computer vision and pattern recognition. Springer, 1–6.

[B18] Fischer A. M. Eid M. De Cecco C. N. Gulsun M. A. Van Assen M. Nance J. W. (2020). Accuracy of an artificial intelligence deep learning algorithm implementing a recurrent neural network with long short-term memory for the automated detection of calcified plaques from coronary computed tomography angiography. J. Thorac. imaging 35, S49-S57–S57. 10.1097/RTI.0000000000000491 32168163

[B19] Garrone P. Biondi-Zoccai G. Salvetti I. Sina N. Sheiban I. Stella P. R. (2009). Quantitative coronary angiography in the current era: principles and applications. J. interventional Cardiol. 22, 527–536. 10.1111/j.1540-8183.2009.00491.x 19627430

[B20] Gu A. Dao T. (2023). Mamba: linear-time sequence modeling with selective state spaces. arXiv Prepr. arXiv:2312.00752. 10.48550/arXiv.2312.00752

[B21] Hammami M. Friboulet D. Kechichian R. (2020). “Cycle GAN-based data augmentation for multi-organ detection in CT images via YOLO,” in 2020 IEEE international conference on image processing (ICIP), Abu Dhabi, United Arab Emirates, 25-28 October 2020 (IEEE), 390–393.

[B22] Han T. Ai D. Li X. Fan J. Song H. Wang Y. (2023). Coronary artery stenosis detection via proposal-shifted spatial-temporal transformer in X-ray angiography. Comput. Biol. Med. 153, 106546. 10.1016/j.compbiomed.2023.106546 36641935

[B23] Hao J. He L. Hung K. F. (2024a). T-mamba: frequency-enhanced gated long-range dependency for tooth 3d cbct segmentation. arXiv Prepr. arXiv:2404.01065. 10.48550/arXiv.2404.01065

[B24] Hao S. Li X. Peng W. Fan Z. Ji Z. Ganchev I. (2024b). YOLO-CXR: a novel detection network for locating multiple small lesions in chest X-ray images. IEEE Access 12, 156003–156019. 10.1109/access.2024.3482102

[B25] He K. Gkioxari G. Dollár P. Girshick R. (2017). Mask r-cnn. Proc. IEEE Int. Conf. Comput. Vis., 2961–2969. 10.1109/ICCV.2017.322

[B26] Ji Z. Zhao J. Liu J. Zeng X. Zhang H. Zhang X. (2023). ELCT-YOLO: an efficient one-stage model for automatic lung tumor detection based on CT images. Mathematics 11, 2344. 10.3390/math11102344

[B27] Jiménez-Partinen A. Molina-Cabello M. A. Thurnhofer-Hemsi K. Palomo E. J. Rodríguez-Capitán J. Molina-Ramos A. I. (2024). CADICA: a new dataset for coronary artery disease detection by using invasive coronary angiography. Expert Syst. 41. 10.1111/exsy.13708

[B28] Jocher G. Chaurasia A. Stoken A. Borovec J. Kwon Y. Michael K. (2022). Ultralytics/yolov5: v6. 2-yolov5 classification models, apple m1, reproducibility, clearml and deci. ai integrations. Switzerland: Zenodo.

[B29] Kishore A. N. Jayanthi V. (2019). Automatic stenosis grading system for diagnosing coronary artery disease using coronary angiogram. Int. J. Biomed. Eng. Technol. 31, 260–277. 10.1504/ijbet.2019.102974

[B30] Li X. Ai D. Song H. Fan J. Fu T. Xiao D. (2024). STQD-det: spatio-temporal quantum diffusion model for real-time coronary stenosis detection in X-ray angiography. IEEE Trans. Pattern Analysis Mach. Intell. 46, 9908–9920. 10.1109/TPAMI.2024.3430839 39024088

[B31] Liu J. Yang H. Zhou H. Y. Xi Y. Yu L. Li C. (2024). “Swin-umamba: mamba-based unet with imagenet-based pretraining,” in International conference on medical image computing and computer-assisted intervention. Springer, 615–625.

[B32] Liu K. (2022). Stbi-yolo: a real-time object detection method for lung nodule recognition. IEEE Access 10, 75385–75394. 10.1109/access.2022.3192034

[B33] Lu G. Ye W. Ou J. Li X. Tan Z. Li T. (2021). Coronary computed tomography angiography assessment of high-risk plaques in predicting acute coronary syndrome. Front. Cardiovasc. Med. 8, 743538. 10.3389/fcvm.2021.743538 34660742 PMC8517134

[B34] Ma J. Li F. Wang B. (2024). U-mamba: enhancing long-range dependency for biomedical image segmentation. arXiv Prepr. arXiv:2401.04722. 10.48550/arXiv.2401.04722

[B35] Mohan N. Vishnukumar S. (2016). “Detection and localization of coronary artery stenotic segments using image processing,” in 2016 International Conference on Emerging Technological Trends (ICETT), Kollam, India, 21-22 October 2016 (IEEE), 1–5.

[B36] Montalbo F. J. P. (2020). A computer-aided diagnosis of brain tumors using a fine-tuned YOLO-based model with transfer learning. KSII Trans. Internet Inf. Syst. (TIIS) 14, 4816–4834. 10.3837/tiis.2020.12.011

[B37] Mortada M. J. Tomassini S. Anbar H. Morettini M. Burattini L. Sbrollini A. (2023). Segmentation of anatomical structures of the left heart from echocardiographic images using Deep Learning. Diagnostics 13, 1683. 10.3390/diagnostics13101683 37238168 PMC10217142

[B38] Ovalle-Magallanes E. Avina-Cervantes J. G. Cruz-Aceves I. Ruiz-Pinales J. (2022). Hybrid classical–quantum Convolutional Neural Network for stenosis detection in X-ray coronary angiography. Expert Syst. Appl. 189, 116112. 10.1016/j.eswa.2021.116112

[B39] Pang K. Ai D. Fang H. Fan J. Song H. Yang J. (2021). Stenosis-DetNet: sequence consistency-based stenosis detection for X-ray coronary angiography. Comput. Med. Imaging Graph. 89, 101900. 10.1016/j.compmedimag.2021.101900 33744790

[B40] Qin C. Jin Y. Zhang Z. Yu H. Tao J. Sun H. (2023). Anti-noise diesel engine misfire diagnosis using a multi-scale CNN-LSTM neural network with denoising module. CAAI Trans. Intell. Technol. 8, 963–986. 10.1049/cit2.12170

[B41] Qin Z. Yang S. Zhong Y. (2024). Hierarchically gated recurrent neural network for sequence modeling. Adv. Neural Inf. Process. Syst. 36. 10.5555/3666122.3667564

[B42] Ragab M. G. Abdulkader S. J. Muneer A. Alqushaibi A. Sumiea E. H. Qureshi R. (2024). A comprehensive systematic review of YOLO for medical object detection (2018 to 2023). IEEE Access 12, 57815–57836. 10.1109/access.2024.3386826

[B43] Rahimi M. Mostafavi M. Arabameri A. (2024). “Automatic detection of brain tumor on MRI images using a YOLO-based algorithm,” 2024 13th Iranian/3rd international machine vision and image processing conference (MVIP), Tehran, Iran, Islamic Republic of, 06-07 March 2024 (IEEE), 1–5.

[B44] Randar S. Shah V. Kulkarni H. Suryawanshi Y. Joshi A. Sawant S. (2024). YOLOv8-based frameworks for liver and tumor segmentation task on LiTS. SN Comput. Sci. 5, 741. 10.1007/s42979-024-03097-5

[B45] Redmon J. Divvala S. Girshick R. Farhadi A. (2016). “You only look once: unified, real-time object detection,” in Proceedings of the IEEE conference on computer vision and pattern recognition, Las Vegas, NV, USA, 27-30 June 2016, 779–788. 10.1109/cvpr.2016.91

[B46] Ren S. He K. Girshick R. Sun J. (2016). Faster R-CNN: towards real-time object detection with region proposal networks. IEEE Trans. pattern analysis Mach. Intell. 39, 1137–1149. 10.1109/TPAMI.2016.2577031 27295650

[B47] Rodrigues D. L. Menezes M. N. Pinto F. J. Oliveira A. L. (2021). Automated detection of coronary artery stenosis in X-ray angiography using deep neural networks. arXiv Prepr. arXiv:2103.02969. 10.48550/arXiv.2103.02969

[B48] Ross T. Y. Dollár G. (2017). “Focal loss for dense object detection,” in proceedings of the IEEE conference on computer vision and pattern recognition, 2980–2988.

[B49] Ruan J. Xiang S., (2024). Vm-unet: vision mamba unet for medical image segmentation. arXiv Prepr. arXiv:2402.02491. 10.48550/arXiv.2402.02491

[B50] Santos C. Aguiar M. Welfer D. Belloni B. (2022). A new approach for detecting fundus lesions using image processing and deep neural network architecture based on YOLO model. Sensors 22, 6441. 10.3390/s22176441 36080898 PMC9460625

[B51] Shi Y. Dong M. Xu C. (2024). Multi-Scale VMamba: hierarchy in hierarchy visual state space model. arXiv Prepr. arXiv:2405.14174.

[B52] Shim J. h. Yu H. Kong K. Kang S. J. (2023). Feedformer: revisiting transformer decoder for efficient semantic segmentation. Proc. AAAI Conf. Artif. Intell. 37, 2263–2271. 10.1609/aaai.v37i2.25321

[B53] Sohan M. Sai Ram T. Reddy R. Venkata C. (2024). “A review on yolov8 and its advancements,” in International conference on data intelligence and cognitive informatics (Springer), 529–545.

[B54] Sun Y. Gong L. Zhang W. Gao B. Li Y. Liu C. (2023). Drivable agricultural road region detection based on pixel-level segmentation with contextual representation augmentation. Agriculture 13, 1736. 10.3390/agriculture13091736

[B55] Tsao C. W. Aday A. W. Almarzooq Z. I. Anderson C. A. Arora P. Avery C. L. (2023). Heart disease and stroke statistics—2023 update: a report from the American Heart Association. Circulation 147, e93–e621. 10.1161/CIR.0000000000001123 36695182 PMC12135016

[B56] Vaswani A. (2017). Attention is all you need. Adv. Neural Inf. Process. Syst. 10.5555/3295222.3295349

[B57] Wan T. Feng H. Tong C. Li D. Qin Z. (2018). Automated identification and grading of coronary artery stenoses with X-ray angiography. Comput. methods programs Biomed. 167, 13–22. 10.1016/j.cmpb.2018.10.013 30501856

[B58] Wang B. Zheng J. Yu J. F. Lin S. Y. Yan S. Y. Zhang L. Y. (2022). Development of artificial intelligence for parathyroid recognition during endoscopic thyroid surgery. Laryngoscope 132, 2516–2523. 10.1002/lary.30173 35638245

[B59] Wang C. J. He C. S. Yan R. X. Liu Y. C. (2023). “Application of MP-YOLO for segmentation and visualization of ovarian ultrasound imaging,” in 2023 IEEE 5th Eurasia Conference on Biomedical Engineering, Healthcare and Sustainability (ECBIOS), Tainan, Taiwan, 02-04 June 2023 (IEEE), 130–132.

[B60] Wang T. Su X. Liang Y. Luo X. Hu X. Xia T. (2024). Integrated deep learning model for automatic detection and classification of stenosis in coronary angiography. Comput. Biol. Chem. 112, 108184. 10.1016/j.compbiolchem.2024.108184 39191164

[B61] Wu W. Zhang J. Xie H. Zhao Y. Zhang S. Gu L. (2020). Automatic detection of coronary artery stenosis by convolutional neural network with temporal constraint. Comput. Biol. Med. 118, 103657. 10.1016/j.compbiomed.2020.103657 32174325

[B62] Xia Z. Pan X. Song S. Li L. E. Huang G. (2022). “Vision transformer with deformable attention,” in Proceedings of the IEEE/CVF conference on computer vision and pattern recognition, 4794–4803.

[B63] Xing Z. Ye T. Yang Y. Liu G. Zhu L. (2024). “Segmamba: long-range sequential modeling mamba for 3d medical image segmentation,” in International conference on medical image computing and computer-assisted intervention. Springer, 578–588. 10.1007/978-3-031-72111-3_54

[B64] Yang Y. Xing Z. Zhu L. (2024). Vivim: a video vision mamba for medical video object segmentation. arXiv Prepr. arXiv:2401.14168. 10.48550/arXiv.2401.14168

[B65] Ye Z. Chen T. Wang F. Zhang H. Zhang L. (2024). P-mamba: marrying perona malik diffusion with mamba for efficient pediatric echocardiographic left ventricular segmentation. arXiv Prepr. arXiv:2402.08506. 10.48550/arXiv.2402.08506 PMC12402081 40890220

[B66] Zeng P. Liu S. He S. Zheng Q. Wu J. Liu Y. (2023). TUSPM-NET: a multi-task model for thyroid ultrasound standard plane recognition and detection of key anatomical structures of the thyroid. Comput. Biol. Med. 163, 107069. 10.1016/j.compbiomed.2023.107069 37364531

[B67] Zhang D. Yang G. Zhao S. Zhang Y. Zhang H. Li S. (2019). “Direct quantification for coronary artery stenosis using multiview learning,” in Medical Image Computing and Computer Assisted Intervention–MICCAI 2019: 22nd International Conference, Shenzhen, China, October 13–17, 2019 (Proceedings, Part II 22. Springer), 449–457.

[B68] Zhang H. Gao Z. Zhang D. Hau W. K. Zhang H. (2022). Progressive perception learning for main coronary segmentation in X-ray angiography. IEEE Trans. Med. Imaging 42, 864–879. 10.1109/TMI.2022.3219126 36327189

[B69] Zhang H. Zhu Y. Wang D. Zhang L. Chen T. Wang Z. (2024). A survey on visual mamba. Appl. Sci. 14, 5683. 10.3390/app14135683

[B70] Zhou J. Zhang B. Yuan X. Lian C. Ji L. Zhang Q. (2023). YOLO-CIR: the network based on YOLO and ConvNeXt for infrared object detection. Infrared Phys. and Technol. 131, 104703. 10.1016/j.infrared.2023.104703

[B71] Zhu L. Liao B. Zhang Q. Wang X. Liu W. Wang X. (2024). Vision mamba: efficient visual representation learning with bidirectional state space model. arXiv Prepr. arXiv:2401.09417. 10.48550/arXiv.2401.09417

